# Investigation of respirable coal mine dust (RCMD) and respirable crystalline silica (RCS) in the U.S. underground and surface coal mines

**DOI:** 10.1038/s41598-022-24745-x

**Published:** 2023-01-31

**Authors:** Elham Rahimi, Younes Shekarian, Naser Shekarian, Pedram Roghanchi

**Affiliations:** 1 Department of Mineral Engineering, New Mexico Institute of Mining and Technology, Socorro, NM USA; 2 John and Willie Leone Family Department of Energy and Mineral Engineering, Earth and Mineral Sciences (EMS) Energy Institute, The Pennsylvania State University, University Park, PA USA; 3 Department of Information System, Business School, University of Colorado Denver, Denver, CO USA

**Keywords:** Diseases, Health care, Medical research

## Abstract

Dust is an inherent byproduct of mining activities that raises notable health and safety concerns. Cumulative inhalation of respirable coal mine dust (RCMD) and respirable crystalline silica (RCS) can lead to obstructive lung diseases. Despite considerable efforts to reduce dust exposure by decreasing the permissible exposure limits (PEL) and improving the monitoring techniques, the rate of mine workers with respiratory diseases is still high. The root causes of the high prevalence of respiratory diseases remain unknown. This study aimed to investigate contributing factors in RCMD and RCS dust concentrations in both surface and underground mines. To this end, a data management approach is performed on MSHA’s database between 1989 and 2018 using SQL data management. In this process, all data were grouped by mine ID, and then, categories of interests were defined to conduct statistical analysis using the generalized estimating equation (GEE) model. The total number of 12,537 and 9050 observations for respirable dust concentration are included, respectively, in the U.S. underground and surface mines. Several variables were defined in four categories of interest including mine type, geographic location, mine size, and coal seam height. Hypotheses were developed for each category based on the research model and were tested using multiple linear regression analysis. The results of the analysis indicate higher RCMD concentration in underground compared to RCS concentration which is found to be relatively higher in surface coal mines. In addition, RCMD concentration is seen to be higher in the Interior region while RCS is higher in the Appalachia region. Moreover, mines of small sizes show lower RCMD and higher RCS concentrations. Finally, thin-seam coal has greater RCMD and RCS concentrations compared to thicker seams in both underground and surface mines. In the end, it is demonstrated that RCMD and RCS concentrations in both surface and underground mines have decreased. Therefore, further research is needed to investigate the efficacy of the current mass-concentration-based monitoring system.

## Introduction

As an inherent byproduct of mining, dust may impose various health and safety issues in mine operations. The term dust is used for solid particles in the air and is defined as airborne particles usually in a size range of 1 to 100 μm^1^. Dust particles of various sizes, typically formed in irregular shapes, generated by mechanical and chemical processes that are estimated to be approximately 3% of the total excavated material^2,3^. The chance of a dust particle to deposit in the human respiratory system depends significantly on the particle size or the aerodynamic diameter^4,5^. Respirable dust generally refers to particles having an aerodynamic diameter less than 10 μm and a median cut-point (d_50_) of 4 μm^6,7^. The United States Mine Safety and Health Administration (MSHA) states that “Any respirable dust in the mine atmosphere is considered respirable coal mine dust to which miners are exposed and, when measured, is counted for determining compliance with the respirable dust standard”^8^.

The primary sources of respirable coal mine dust (RCMD) are found to be coal seam and surrounded rock strata, intake air, diesel exhaust, mining operations, and rock dusting^9–11^. The concentration of dust depends on several factors, including types of activity, the number of free surfaces in the cross-section walls, ventilation, and dust control practices. The duration and total mass of RCMD are the main two factors that regulate the RCMD permissible exposure limit (PEL)in working areas^12^. In the U.S., the PEL for respirable coal dust concentration set to 3 mg/m^3^ in 1970, then reduced to 2 mg/m^3^ in 1972 quartz less than 5% mass of RCMD samples^11,13^. Following these standards, the prevalence of lung diseases and concentrations of coal mine dust declined significantly. However, in the late 1990s resurgence of black lung trigged researchers to investigate the root causes of coal workers’ pneumoconiosis (CWP)^13,14^. MSHA also reduced PELs to 1.5 mg/m^3^ for RCMD and 0.1 mg/m^3^ for RCS in 2014, and sampling was needed for the whole duration of the shift, even more than 8 h. However, many have questioned whether reducing the exposure limits will target the root cause of the problem^11^.

In the late 1990s, despite years of efforts to understand and reduce CWP prevalence from more than 30% in 1970 to less than 4.2%, the level of occurrences among U.S. coal miners increased unexpectedly. Although the connections between coal miners RCMD exposure and respiratory diseases have been studied for decades, the contributing factors that cause the progression of the disease are not yet identified^5,11,15–18^. Several researchers aimed to investigate the effect of RCS, as a major inorganic part of RCMD, on health diseases^19,20^. However, other studies indicated that neither CWP nor progressive massive fibrosis could be credited for RCS until quartz concentration reaches 10%^21–24^.

The effectiveness of MSHA’s recent dust rule (i.e., decreasing the PELs and use of continuous personal dust monitor (CPDM)) will possibly take years to be properly assessed^25^. Nonetheless, the current regulation requires sampling RCMD mass concentration using a CPDM device. However, quartz in RCMD samples is not detectable using the CPDM and MSHA monitor respirable quartz using a personal gravimetric sampler (CMDPSU)^11^. The true dose of environmental and internal exposure, which is based on dust source, concentration, characteristics, and its respiratory deposition, is necessary to be assessed and compared with the data from the mass-concentration-based sampling method. Early 1900’s USA used particle number measurement using konimeter (1916) and Greenburg-Smith impinger (1922) and limit values were expressed as million particles per cubic foot (mppcf, counted under microscope)^26,27^. But it later transpired that particle counting had its disadvantages; (1) very labor intensive and had considerable inter- and intra-observer variabilities, (2) measured concentrations based on the particle count were found not to correlate particularly well with health effects. Rather, it was shown quite early on that the mass concentrations of fine particles provided much better correlations, in particular for certain types of lung diseases such as pneumoconiosis. However, mass concentrationmay not correctly represent the true dose of RCMD received by the coal miner, specifically for diseases caused by particles less than 4 μm. Therefore, the number-concentration-based RCMD samples could be an alternative and ideal index for RCMD dose estimations^28^. There are, however, many factors that contribute to development of lung diseases, including submicron size fractions, particle number, diesel particulate matter (DPM), and other elemental content, which need studies on particle characteristics and effect of particle characteristics on probability of lung deposition^29^.

While the studies mentioned above have explored potential factors that may contribute to the risks of lung diseases among coal workers, many questions remain unanswered^9,11,30^. Has advancement in technology and changes in mining practices caused changes in characteristics and respiratory deposition of respirable dust? What components of dust will cause high rates of CWP and silicosis among coal miners? Does dust mass content represent the true respirable dust dose received by a coal miner? Currently, there is not enough data to answer the above-mentioned questions. High exposures to RCS may increase the chances of a sub-type of silicosis called accelerated or progressive silicosis, while the association with CWP is still unclear^31^. It is evident that more research on the physical and chemical properties of respirable dust is needed to understand the root causes of CWP incidences and its recent resurgence. Characteristics parameters such as particle size distribution (particularly in the smaller fractions enriched with minerals), mineral composition, trace element presence, particle shape, and angularity are critical parameters that need to be examined closely.

This study aims to provide a better insight into the root causes of lung disease’s resurgence among coal workers by investigating respirable dust concentrations in both U.S. underground and surface coal mines. For this purpose, statistical analysis is performed on MSHA’s database for 30 years (1989–2018) to investigate the effect of various mining parameters (mine type, geographic location, mine size, seam height) on RCMD and RCS concentration in a multivariable model. This statistical approach is needed to ensure that data are appropriately understood, and those apparent relationships are meaningful (i.e., "significant") and not only incidental. This technique that uses numbers, attempts to eliminate any biases while analyzing data. There are different ways to fit a straight line to the data when trying to uncover the trends, such as regression analysis, which is a statistical technique for studying linear relationships. The potential reasons for dust exposure in coal mines were investigated by testing developed hypotheses on defined variables for RCMD and RCS based on the research model. In this study, the data management approach is also explained in detail, and variables of interest are selected in different categories. The categories and the description of each variable were presented in Table 1. RCMD and RCS categories are the dependent variables that are of interest to investigate any relationship with the independent variables. Then, the observations by each category and corresponding variables are discussed, and a descriptive analysis of RCMD and RCS data for each variable with the correlation coefficient between variables is presented. According to the observation for each category, hypotheses are developed separately for RCMD and RCS samples in both underground and surface. Then, a multiple linear regression analysis is conducted to uncover any patterns or trends in RCMD and RCS concentrations. The result would significantly help future studies on conducting research works to perform detailed experiments on respirable dust exposure and deposition in the human lung. These recommended investigations would provide the basic knowledge on finding roots of lung disease resurgence among the United States coal workers.

**Table 1:** The description of defined categories and subcategories of independent variables for RCMD and RCS as dependent variables of interest.

Category	Variable	Description	Type
RCMD	–	Respirable coal mine dust measurements in mg/m^3^	Dependent
RCS	–	Respirable crystalline silica measurements in % mass of RCMD samples	Dependent
Mine type	Surface	If the mine type is surface in the United States (1 = yes; 0 = no)	Independent
	Underground	If the mine type is underground in (1 = yes; 0 = no)	Independent
Geographic location	Appalachia	West Virginia, Kentucky, Pennsylvania, Virginia, Alabama, Tennessee, Florida, Georgia, New York, North Carolina, Maryland, South Carolina, New Jersey, Puerto Rico, Vermont, Massachusetts, Mississippi	Independent
	Western	California, Nevada, Arizona, Colorado, Wyoming, Utah, Montana, Idaho, Washington, South Dakota, Alaska, Oregon, North Dakota, Hawaii, Northern Mariana Islands	Independent
	Interior	Illinois, Texas, Ohio, Indiana, Missouri, Michigan, Minnesota, New Mexico, Oklahoma, Wisconsin, Arkansas, Kansas, Iowa, Louisiana, Nebraska	Independent
Mine size	Small	The average number of employees per each coal mine is less than 50 (1 = yes; 0 = no) (both underground and surface)	Independent
	Medium	The average number of employees per each coal mine is between 50 and 100 (1 = yes; 0 = no) (both underground and surface)	Independent
	Large	The average number of employees per each coal mine is more than 100 (1 = yes; 0 = no) (both underground and surface)	Independent
Coal seam height	Thickness ≤ 40 in.	If the average thickness of the coal seam in the mine is less than 40 in. (1 = yes; 0 = no)	Independent
	40 < height ≤ 75 (in.)	If the average thickness of the coal seam in the mine is between 40–75 in. (1 = yes; 0 = no) (only underground)	Independent
	Thickness > 75 in.	If the average thickness of the coal seam in the mine is more than 75 in. (1 = yes; 0 = no) (only underground)	Independent

## Material and methods

**Figure 1:**
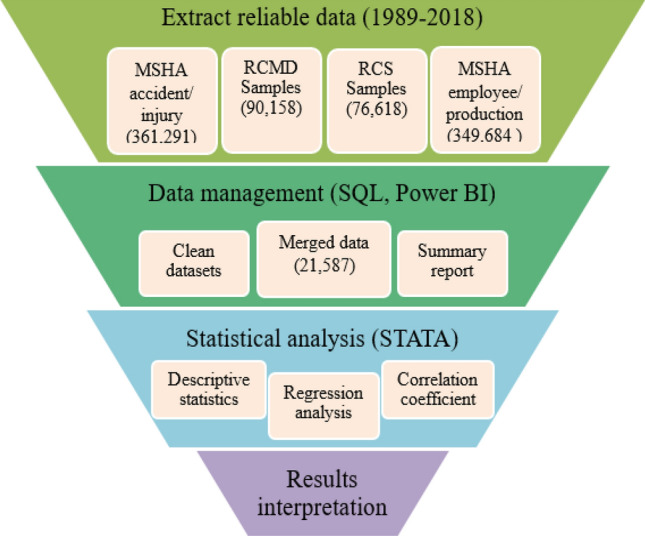
Summary of the data source and methodology for statistical analysis. The first step is to extract reliable data from different data sources. Second, the data management approach is applied to the extracted data, that this step includes cleaning the duplicate and invalid data, merging data based on the mine-ID or cassette numbers of samples, and providing the report of information included with data sets. The third step indicates a statistical analysis of the extracted data, which finally concludes with the interpretation of results.

The datasets were collected from the MSHA’s library (https://arlweb.msha.gov/OpenGovernmentData/OGIMSHA.asp). The datasets include accident injury/illnesses (361,291 records), employment/production (349,684 records), respirable crystalline silica (RCS) samples (76,618 records), and respirable coal mine dust (RCMD) samples (90,158 records) (Fig. 1). RCMD and RCS samples were merged based on the cassette number. The information related to subunit, employee, coal seam height, county code, and lung disease records were taken from the rest of the datasets. All information from different data sets are merged by mine-ID using Structural Query Language (SQL) Server Management Studio. SQL provides a convenient environment to define categories of interests and group data sets to provide a summary of consistent data sets. Categories by different variables were coded in SQL, including mine type, geographic location, mine size, and coal seam height^32^. According to the primary visualization of data, variables of interest were assumed to have a relationship with dust concentration. The description of variables and defined categories are provided in Fig. 1.

Finally, a summary of data is directly imported to Microsoft Office Power BI visualization to export the observations report. These reports were used in statistical analysis using STATA to provide descriptive statistics of variables, correlation, and regression analysis.

The raw data from the MSHA’s library is used for two dependent categories of RCMD and RCS. The category of geographic location is defined by Energy Information Administration (EIA) classification and referred to the state in which the mine site is located, including Western, Interior, and Appalachia regions^33^. The category of mine type is available in MSHA’s library as a subunit. In this study, subunits other than underground and surface were grouped as “other” variables. The thickness of coal seams is also provided by MSHA’s library in inches, classified into three classes of less than 40 inches, between 40 and 75 in., and greater than 75 in. For the mine size category, MSHA’s employee/production data set is used to define groups of mine sizes based on the number of the employees, including less than 50 employees (small), between 50 and 100 employees (medium), and greater than 100 employees (large). It worth mentioning that none of these data sets include all the needed information. For instance, the RCS data sets lacked the subunit information and geographic location of the sample taken. The mine-IDs were merged with RCMD sample datasets (with the subunit information) in order to provide a consistent database. Then, the mine-IDs were merged with MSHA accident/injury and MSHA employee/production datasets to include the mining parameters and additional information related to each sample.

### RCMD and RCS samples

The average concentrations of RCMD (mg/m^3^) and RCS (mass percentage of RCMD sample) were determined for each year. The samples from the surface and underground mines were separated and number of samples that exceeded the PEL over the years is provided in Table 2. The average RCMD concentrations were consistently below the PEL in underground mines, and samples that exceeded the limit were non-significant. However, the average concentrations of RCS samples were slightly above the PEL, and the number of samples exceeding the PEL is considerable. A similar trend for RCMD samples in the surface mines is observed. However, the RCS samples’ average concentrations were considerably above the PEL, and the majority of samples exceeded the PEL. It can be shown that regulations efficiently reduced RCMD exposure in both underground and surface mines. In other words, the number of RCMD samples that exceeded the PEL is almost zero in the most recent years. However, an important constituent of RCMD exposure as RCS has been overlooked. The average of RCS samples exceeded the PEL in this time period remained as 55.7% and 86.3% of all samples taken, respectively, in underground and surface mines.

**Table 2:** The total number of RCMD and RCS samples per year and percent of samples exceeded the PEL in both underground and surface mines.

Year	Underground						Surface					
	RCMD			RCS			RCMD			RCS		
	No sample	> PEL	(% Sample) > PEL	No sample	> PEL	(%Sample) > PEL	No sample	> PEL	(% Sample) > PEL	No sample	> PEL	(% Sample) > PEL
1989	225	51	22.7	240	146	60.8	39	6	15.4	36	32	88.9
1990	719	141	19.6	737	470	63.8	168	20	11.9	165	143	86.7
1991	965	168	17.4	734	467	63.6	89	11	12.4	88	69	78.4
1992	734	154	21	773	484	62.6	137	17	12.4	141	130	92.2
1993	628	97	15.4	667	407	61	221	31	14	228	207	90.8
1994	553	73	13.2	598	360	60.2	205	15	7.3	212	179	84.4
1995	621	36	5.8	669	455	68	301	9	3	308	283	91.9
1996	619	53	8.6	661	454	68.7	320	3	0.9	331	305	92.1
1997	637	37	5.8	680	447	65.7	353	3	0.8	365	349	95.6
1998	683	50	7.3	726	533	73.4	353	6	1.7	367	339	92.4
1999	614	25	4.1	651	459	70.5	361	4	1.1	377	360	95.5
2000	562	24	4.3	611	384	62.8	257	4	1.6	261	241	92.3
2001	630	7	1.1	577	362	62.7	575	1	0.2	201	177	88.1
2002	578	9	1.6	495	263	53.1	571	3	0.5	141	117	83
2003	497	6	1.2	417	224	53.7	532	0	0	120	99	82.5
2004	521	4	0.8	470	276	58.7	594	1	0.2	139	115	82.7
2005	527	6	1.1	463	282	60.9	610	1	0.2	135	122	90.4
2006	519	8	1.5	450	261	58	559	3	0.5	128	118	92.2
2007	495	3	0.6	408	244	59.8	551	0	0	131	117	89.3
2008	522	6	1.1	447	259	57.9	681	1	0.1	124	105	84.7
2009	380	1	0.3	397	224	56.4	673	2	0.3	127	115	90.6
2010	73	1	1.4	385	199	51.7	631	0	0	98	88	89.8
2011	71	0	0	393	195	49.6	650	1	0.2	93	76	81.7
2012	80	0	0	351	154	43.9	599	2	0.3	74	61	82.4
2013	66	0	0	281	114	40.6	538	1	0.2	51	42	82.4
2014	46	0	0	293	98	33.4	499	0	0	92	72	78.3
2015	40	0	0	279	93	33.3	444	1	0.2	162	120	74.1
2016	216	0	0	221	76	34.4	530	0	0	145	117	80.7
2017	233	1	0.4	222	89	40.1	501	0	0	182	139	76.4
2018	270	0	0	221	90	40.7	492	1	0.2	158	126	79.7
Total	13,324	961	7.2	14,517	8,569	59.0	13,034	147	1.1	5,180	4,563	88.1

### Developed hypothesis based on the dust concentrations in underground and surface coal mines

Given the number of 21,587 observations based on mine-ID, several variables within the database were investigated. Among these variables, mine type, geographic location, coal seam height, and mine size were selected to perform analysis on any probable relation with RCMD and RCS concentration in the U.S. underground and surface coal mines between 1989 and 2018.

#### Mine type

Underground mining is associated with higher dust concentrations in comparison with surface mines. However, a previous study showed that surface coal mines generate more air pollution containing respirable particulate matter than underground^34^. A few studies investigated the coal dust and silica dust concentrations in U.S. coal mines. Doney et al.^35,36^ investigated the mass concentration of RCMD and RCS percentages using the MSHA database between 1982 and 2018. The result of 681,497 RCMD and 210,944 RCS samples in underground mines showed an average of 0.55 mg/m^3^ and 0.038 mg/m^3^ for RCMD and RCS concentrations, respectively. However, in surface mines, based on 288,705 RCMD and 54,040 RCS samples, these averages are 0.17 mg/m^3^ and 0.02 mg/m^3^, respectively. Therefore, the number of RCMD samples exceeding the PEL in the underground is three times greater than in surface mines. Therefore, two hypotheses is provided to test if the mine type is a contributing factor in dust concentrations.

(H1)_R_: Workers in underground coal mines are exposed to higher RCMD concentrations than the surface.

(H1)_Q_: Workers in surface coal mines are exposed to higher RCS concentrations than underground.

#### Geographic location

The geological location is the dominant factor in coal formation, including coal rank, roof/floor layers, and constituent elements. Previous studies highlighted that central Appalachia reported lower dust concentrations compared to the rest of the regions^35,36^. For example, dust from mid-central and south-central Appalachia mines had higher percentages of aluminosilicate and silica particles than dust from northern Appalachia mines^37^. The finding of previous studies provides information to develop the hypothesis related to the geographic location, indicating that dust concentrations, including RCMD and RCS particles, have a different distribution.

(H2)_R_: Geographic location of a mine contributes to the level of RCMD concentrations.

(H2)_Q_: Geographic location of a mine contributes to the level of RCS concentrations.

#### Mine size

In recent studies, mine size is defined based on the number of employees working at the mine site. Blackely et al.^13^ investigated the prevalence of lung disease as a mine size function. The result showed that small coal mines are accused of more dust exposures, causing a greater lung issue incidence. Antao et al.^38^ also indicated a greater RCMD dust concentration in smaller mine sites^38^. However, other researchers claimed a negative correlation between mine size and lung disease prevalence^39,40^. These contradictions in previous studies’ findings provided an idea of developing hypotheses based on the mine size with RCMD and RCS concentrations.

(H3)_R_: RCMD concentration is higher in large mines.

(H3)_Q_: RCS dust concentration is higher in small mines.

#### Coal seam thickness

Previously, it was discussed that geographical location could be a significant factor contributing to the RCMD and RCS concentrations due to the variations in coal seam thickness and surrounding layers. The layer of coal plays a substantial role in dust generation; the thicker the coal seam causes higher RCMD concentration. On the other hand, the thinner coal seam leads to more crushing of the surrounding layers, which means greater RCS concentrations^37^. In a survey conducted by Blackley^13^, lung diseases were prevalent and more severe in small mines with a thin layer of coal seams^13^. Further to previous studies, the hypothesis related to coal seam height is developed to determine the relationship between coal thickness and concentration of RCMD and RCS in different mines.

(H4)_R_: The lower thickness of the coal seam produces greater RCMD dust concentration.

(H4)_Q_: The higher thickness of the coal seam causes greater RCS dust concentration.

### Consent to participate

The authors have consent to participate.

## Research model using generalized estimating equation (GEE)

This study developed a research model for testing hypotheses that indicates the dependent variables of RCMD and RCS dust concentrations, and four presented hypotheses on mine type (H1), geographic location (H2), mine size (H3), and coal seam height (H4) that were tested separately for both RCMD and RCS dust samples.

### Descriptive statistics

A total of 21,587 mine-year observations were included in the regression analysis. The number of 12,537 observations is related to RCMD samples, including 6428 observations for underground and 6109 observations for surface mines. Also, the number of 9050 is related to RCS samples, including 6392 for underground and 2658 for surface mines. These observations were obtained by the number of groups (mine-ID) multiplied by the average of observations per group. For example, the number of groups in underground RCMD is 1404 multiplied by average observations per group of 4.57 gives the number of 6428 observations.

- Observations for RCMD in underground: 1404 (mine-ID) * 4.578 (avg.) = 6428
- Observations for RCMD in surface: 1364 (mine-ID) * 4.478 (avg.) = 6109
- Observations for RCS in underground: 1410 (mine-ID) * 4.533 (avg.) = 6392
- Observations for RCS in surface: 939 (mine-ID) * 2.830 (avg.) = 2658.

The descriptive statistics of RCMD (Tables 3) and RCS (Table 4) samples for each category are provided separately for underground and surface mines. Descriptive statistics include average, standard deviation, minimum, and maximum of data for each variable. As described, RCMD and RCS are the dependent variables. The rest of the variables are considered as independent and dummy variables. A dummy variable is one that only takes the value of 0 or 1 for statistics, especially in regression studies, to indicate the absence or presence of a categorical effect that may affect the results. For instance, in Table 3, the three mean of variables Appalachia, Western, and Interior in the underground are 0.90, 0.04, and 0.06, respectively, which represents 90 percent of geographic location data is related to the Appalachia region, and 4% and 6% pertaining to Western and Interior regions, respectively.

**Table 3:** Descriptive statistics of RCMD samples in each category and subcategory for independent variables in both underground and surface mines.

Variables		Underground observations (6428)				Surface observations (6109)			
		Mean	Std. dev	Min	Max	Mean	Std. dev	Min	Max
Dust type	RCMD (mg/m^3^)	1.05	1.09	0.02	44.4	0.40	0.67	0.00	31.1
Geographical location	Appalachia	0.90	0.30	0	1	0.82	0.38	0	1
	Western	0.04	0.19	0	1	0.06	0.25	0	1
	Interior	0.06	0.24	0	1	0.11	0.31	0	1
Mine size	Small	0.71	0.45	0	1	0.78	0.41	0	1
	Medium	0.12	0.33	0	1	0.13	0.34	0	1
	Large	0.17	0.37	0	1	0.09	0.29	0	1
Seam height	Seam height1	0.35	0.48	0	1	0.58	0.49	0	1
	Seam height2	0.52	0.50	0	1	0.31	0.46	0	1
	Seam height3	0.14	0.34	0	1	0.11	0.31	0	1

**Table 4:** Descriptive statistics of RCS samples in each category and subcategory for independent variables in both underground and surface mines.

Variables		Underground observation (6392)				Surface observation (2658)			
		Mean	Std. dev	Min	Max	Mean	Std. dev	Min	Max
Dust type	RCS (%)	5.97	3.74	0.1	48.3	13.48	9.31	0.1	99.9
Geographical location	Appalachia	0.87	0.34	0	1	0.83	0.37	0	1
	Western	0.04	0.20	0	1	0.06	0.23	0	1
	Interior	0.09	0.29	0	1	0.11	0.31	0	1
Mine size	Small	0.63	0.48	0	1	0.73	0.44	0	1
	Medium	0.13	0.34	0	1	0.18	0.38	0	1
	Large	0.24	0.43	0	1	0.09	0.29	0	1
Seam height	Seam height1	0.31	0.46	0	1	0.58	0.49	0	1
	Seam height2	0.54	0.50	0	1	0.31	0.46	0	1
	Seam height3	0.16	0.36	0	1	0.12	0.32	0	1

According to descriptive statistics, the majority of RCMD samples were collected from the Appalachia region both in the underground (90%) and surface (82%) mines. Moreover, greater number of RCMD samples accounts for small mines in both underground (71%) and surface (78%) in the mine size category. However, the distribution of RCMD samples based on coal seam height has been observed to be distributed evenly in both underground and surface mines. Coal seams with thickness greater than 75 in. seem to have fewer samples than other seam height groups.

In Table 4, in geographic location category, most RCS samples were taken from the Appalachia region both in underground (87%) and surface (83%). Moreover, greater number of RCS samples accounts for small mines in both underground (63%) and surface (73%) in the mine size category. However, the distribution of RCS samples based on coal seam height has been observed to be more proportional in both underground and surface mines. Coal seams with thickness greater than 75 in. seem to have fewer samples compared to other seam height groups (Table 4).

**Figure 2:**
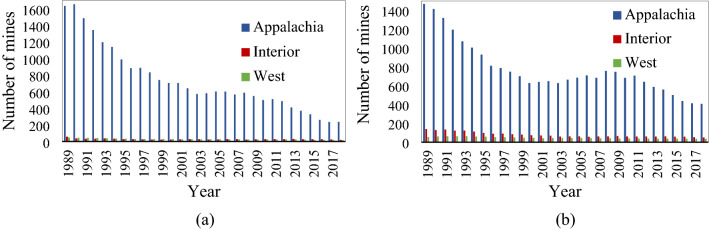
Number of underground (**a**) and surface (**b**) coal mines included in this study.

Based on the descriptive, it can be inferred that the main focus of dust sampling is Appalachia region. Since the number of coal mines is significantly higher in Appalachia than other regions (Fig. 2a,b), it can be deduced that a significant number of these mines are in small sizes, which samples were taken. Moreover, underground coal seams are more with a thickness of between 40 and 75 in., while in surface mines, coal seam heights are less than 40 in.

### Correlation analysis

A summary of the relatively single variable, frequency, and correlation coefficients is the standard technique to investigate in statistical analysis^41–43^. The results of the correlation analysis between variables are most significant, with 99% confidence, in underground mines (see Table 5, different significant levels are highlighted in different levels of green color). Underground mines in Appalachia were correlated with small sizes (0.358), while in Interior (0.380) and Western (0.200) were correlated with large sizes. Also, Appalachia mines have coal thickness less than 40 in. (0.241) while in Western (0.376), and Interior (0.184) regions, coal seams have a thickness greater than 75 in. Generally, it can be inferred that small mines have coal seams less than 40 in. (0.381), medium mines have coal seams with a thickness between 40–75 in. (0.165), and large mines have coal seams with greater than 75 in. thickness (0.301).

**Table 5:**
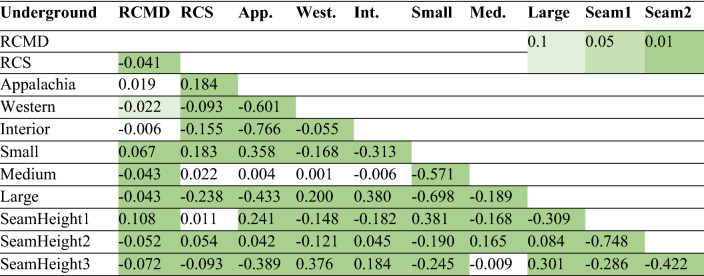
Correlation of variables for underground coal mines in the U.S., 1989–2018.

Numbers imply the significance of correlation coefficient at levels: p < 0.1, p < 0.05, p < 0.01.

Similar results were observed for surface mines (i.e., Appalachian mines were correlated with small sizes (0.318), and Interior region (0.073) and Western region (0.651) were correlated with large mine sizes) (Table 6). Coal seams with a thickness of less than 40 in. were significant in Appalachian region (0.071). On the other hand, coal seams with a thickness greater than 75 in. in Western (0.567) and between 40 and 75 in. in interior (0.028) region were significant. Likewise, it can be inferred that small mines have coal seams less than 40 in. (0.226), and large mines have coal seams with greater than 75 in. thickness (0.378).

**Table 6:**
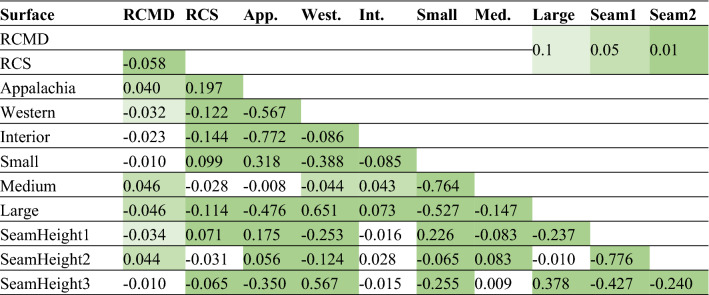
Correlation of variables for surface coal mines in the U.S., 1989–2018. Numbers imply the significance of correlation coefficient at levels: p < 0.1, p < 0.05, p < 0.01.

### Regression analysis

Typically, regression analysis is performed to describe the relationships between a set of independent variables (i.e., mine type, geographic location, mine size, coal seam height) and the dependent variables (i.e., RCMD and RCS concentrations). Regression analyses generate a regression equation where the coefficients reflect each independent variable's relationship with the dependent variable. For each possible setting of the independent variables, there is a separate population of response values in a regression model^44^. While the dependent variable's mean value can be modeled by many functions depending on one or more independent variables, the choice of the proper regression model usually depends on the type of dependent variable data and the model type^45,46^. According to the nature of the dataset, which is an unbalanced panel data (an unbalanced panel data is a dataset in which at least one panel member is not observed every period, for example, the number of RCMD and RCS samples were not consistent over 30 years’ period), an estimation method to predict the relationship between explanatory variables across the dust concentration (RCMD and RCS) is needed. Different regression models have been tried on the panel data to determine the best fit based on the nature of variables which GEE is selected to proceed with further analysis^42,46^. GEE estimates the marginal effect of covariates averaged across units^47,48^. This study can be interpreted as the overall effect of mine type, geographic location, mine size, and coal seam thickness on RCMD and RCS concentration, which has significant relation with lung diseases among coal miners. GEE is widely used for panel estimation^49,50^. The GEE is used to investigate the variation in RCMD and RCS concentrations per mine-year across 30-years unbalanced panel data.

Using SQL Server management studio provides 12,537 number of observations for RCMD and 9050 observations for RCS. The following equation is used as the best fit for the data:

1 $${\text{Y}}_{{{\text{i}},{\text{ t}}}} = \,\upbeta {\text{X}}_{{{\text{i}},{\text{ t}}}} + {\text{ u}}_{{{\text{i}},{\text{ t}}}} + \,\upvarepsilon _{{{\text{i}},{\text{ t}}}}$$

where Y represents the number of RCMD and RCS dust samples; X is a vector of mine-related information, β is the coefficients (values obtained for each variable in GEE result tables); i stand for mine-ID, t indicates the year; therefore, i.t show the effect of the variable by mine-year; u_i_ demonstrates mine-specific unobserved heterogeneity (i.e., factors constant over time but unobserved to the econometrician), and ɛ is the error term (e.g., observation-specific error)^44,45^.

The Variance Inflation Factors (VIF) measures the inflation in the variances of the parameter estimates due to collinearities that exist among the regressor (independent) variables^50^. All VIF values are below the recommended threshold of 10, indicating that multicollinearity is not a concern. Each of the VIF scores for the dataset met this requirement (mean score of 1.47). Table 7 shows the VIF value for variables. This study also uses a robust regression procedure to improve the validity of the baseline model. Robust regression provides an alternative to the least square regression once outliers contaminate data. To detect influential observations, robust regression can also be helpful. Also, if the random variation in the data is not normal or where the data includes large outliers, robust regression models help filter linear relationships^51,52^. The advantage of using robust regression is achieving accurate estimation results and controlling the outlier data.

**Table 7:** The value of variables for VIF test.

Variable	VIF
Underground	1.25
Surface	1.16
Medium mine size	1.06
Large mine size	1.26
Interior region	1.04
Western region	1.17
Seam height ≤ 40	3.25
Seam height > 40 and ≤ 75	3.08

One of the main assumptions of GEE is the homogeneity of variance of the residuals. In other words, the variance of residuals is approximately equal for all predicted dependent variables. It makes the regression prediction unbiased, consistent, and effective. If the model is well-fitted, there should be no pattern to the residuals plotted against the fitted values^40^. The existence of homoscedasticity is tested using Breusch-Pagan. The p-value is statistically significant at 0.01 level and Chi square is 687,934.5. Therefore, the null hypothesis, which is the existence of homoscedasticity, is rejected and the robust standard error is used to account for the heteroscedasticity^41^.

## Results and discussion

Table 8 and 9 show the results of the GEE analysis on the RCMD and RCS dust samples. It shows that coal workers in underground mines are at a higher risk of RCMD exposure than surface miners (Table 8, mine type category, *β* = 0.697, *p* < 0.01). Therefore, H1 is supported for RCMD. However, as for RCS dust concentration, underground mines show a significant negative value, which means surface mines have higher RCS concentration (Table 9, mine type category, *β* = −0.783, *p* < 0.01). This supported H1 for RCS.

**Table 8:** GEE estimation results for RCMD independent variables in underground and surface mines.

Category	Variables	Underground	Surface
Mine type	Surface (reference)		
	Underground	0.697*** (0.029)	
Geographic location	Western (reference)		
	Appalachia	− 0.003 (0.043)	0.022 (0.112)
	Interior	0.212*** (0.055)	−0.046 (0.118)
Mine size	Small (reference)		
	Medium	0.025 (0.033)	0.147** (0.071)
	Large	0.097*** (0.036)	0.023 (0.068)
Coal seam thickness	Seam height > 75 (reference)		
	Seam height ≤ 40	0.147*** (0.041)	0.024 (0.053)
	40 < Seam height ≤ 75	0.040 (0.034)	0.100** (0.064)
	Constant	2.742*** (0.051)	0.574*** (0.297)
	Observations	6428	6109
	Year	1989–2018	1989–2018
	Wald Chi^2^	1528.57***	2879.14***

Standard errors in parentheses, and significant levels: ***p < 0.01, **p < 0.05, *p < 0.1.

**Table 9:** GEE estimation results for RCS independent variables in underground and surface mines.

Category	Variables	Underground	Surface
Mine type	Surface (reference)		
	Underground	− 0.783*** (0.022)	
Geographic location	Western (reference)		
	Appalachia	0.395*** (0.090)	0.365*** (0.117)
	Interior	0.044 (0.096)	0.057 (0.120)
Mine size	Small (reference)		
	Medium	− 0.040 (0.024)	- 0.119*** (0.035)
	Large	− 0.226*** (0.032)	− 0.133** (0.064)
Coal seam thickness	Seam height > 75 (reference)		
	Seam height ≤ 40	− 0.125*** (0.036)	− 0.039 (0.065)
	40 < Seam height ≤ 75	− 0.025 (0.028)	− 0.100* (0.069)
	Constant	1.683*** (0.102)	2.741*** (0.196)
	Observations	6392	2658
	Year	1989–2018	1989–2018
	Wald Chi^2^	347.57***	197.07***

Standard errors in parentheses, and significant levels: ***p < 0.01, **p < 0.05, *p < 0.1.

Following the results provided in Table 8, as hypothesized in H2, there is a significant positive value for Interior geographic location compared to the Western region in underground (geographic location category, *β* = 0.212, *p* < 0.01). However, based on the available data, conclusions cannot be presented for mines in the Appalachian region (geographic location category, *β* = −0.003, *insignificant p-value*). Likewise, the result for RCMD concentration by geographic location in the surface mines is not significant. Therefore, H2 is supported (only Interior vs. Western) for RCMD concentrations in underground mines, and not supported in surface mines. On the other hand, in the case of RCS concentrations, both underground and surface mines in Appalachia show a significant relationship compared to the Western region (Table 9, geographic location category, *β* = 0.395 (underground), *β* = 0.365 (surface), *p* < 0.01). Therefore, H2 is also supported (only Appalachia vs. Western) for RCS concentration, both underground and surface mines.

The hypothesis testing results for the mine size category show that underground large mines have higher RCMD concentrations compared to the smaller operations (Table 8, mine size category, *β* = 0.097, *p* < 0.01). But when looking at the coefficient, it is relatively small. In surface mines, RCMD concentrations are higher in medium size (*β* = 0.147, *p* < 0.05). This supported H3 for higher RCMD concentrations in large mine sizes both underground (only large vs. small sizes) and surface mines (only medium vs. small). In the case of RCS concentration in underground mines, the large mine size category shows lower concentrations (Table 9, mine size category, *β* = −0.226, *p* < 0.01). However, in surface mines, medium and large mines show lower RCS concentrations compared to small mines (*β* = −0.119 (Medium), *p* < 0.01, *β* = −0.133 (Large), *p* < 0.05). Therefore, H3 is supported for higher RCS concentrations in small mine sizes both in underground (only large vs. small size) and surface mines.

The coal seam height category results show that RCMD in underground mines with seam height less than 40 in. is relatively higher than the mines with seam thicknesses greater than 75 in. (Table 8, coal seam height category, *β* = 0.147, *p* < 0.01). The surface mines with coal seam height between 40 and 75 in. have higher RCMD concentrations compared to the mine with seam thicknesses greater than 75 in. (*β* = 0.100, *p* < 0.05). Therefore, H4 is supported for RCMD concentrations in underground mines that there is a relationship with coal seam height (only seam height ≤ 40 vs. seam height > 75 in.). Also, H4 is supported in surface mines (only in seam height between 40 and 75 vs. seam height greater than 75 in.). Table 9 represents the results of hypothesis testing for RCS concentration. The results show that the underground coal mines with seam thicknesses less than 40 in. in underground mines have lower RCS concentrations (*β* = −0.125, *p* < 0.01) compared to seam height greater than 75 in. Similarly, in surface mines, coal seams with thicknesses between 40 and 75 in. have a lower RCS concentration (*β* = −0.100, *p* < 0.1). According to these results, H4 for RCS samples is also supported in underground mines (only in seam height ≤ 40 vs. seam height > 75 in.) and surface mines (only in seams between 40 and 75 vs. seam height > 75 in.).

The analysis of worker’s dust exposure is a complex process since there is insufficient information about how sampling is conducted in each mine type and whether there are any differences in the samples taken from an underground or surface mine. Moreover, the investigation of contributing factors is complicated since each variable directly or indirectly relates to other factors. For example, when discussing the effect of mine size on dust concentration, it is a vivid fact that the mine size may also depend on the coal seam thickness, although the mine size is defined by the number of employees working on the mine site. Using the correlation tables in this study also shows that mines in Appalachia are dominantly small sizes with coal seam thickness less than 40 in. in both underground and surface mine types. On the other hand, Western coal mines are usually large sizes with coal seam thickness greater than 75 in. in both mine type categories. Interior region mines are also large sizes in both mine types while with seams greater than 75 in. in the underground and between 40 and 75 in. in the surface mines.

The regression analysis showed significant results about the relationships between dependent variables (RCMD and RCS) and independent variables (mine type, geographic location, mine size, and coal seam height). Hypothesis 1 related to mine type indicated workers in underground mines are exposed to higher RCMD concentration, however, in surface mines, workers are exposed to higher RCS concentration. Hypothesis 2 investigated the contribution of geographic location in respirable dust concentration, indicating that geographic location (only Interior vs. Western) contributed to RCMD concentration for underground mines. However, this hypothesis is not supported for RCMD concentration in surface mines. The results of this hypothesis for RCS concentration supported the contribution of geographic location (only Appalachia vs. Western) with RCS concentration for both underground and surface mines.

Hypothesis 3 on the relationship between mine size and respirable dust concentration showed higher RCMD concentrations in large mine sizes (only large vs. small mine sizes) in underground and surface (only medium vs. small) coal mines. Furthermore, it showed higher RCS concentrations in small mine sizes (only large vs. small mine size) in underground mines, as well as in surface mines (for all three mine sizes). Hypothesis 4 on the relation of coal seam thickness with respirable dust concentration showed a reverse relation between RCMD concentrations with seam thickness (only seam height ≤ 40 in. vs. seam height > 75 in.) in underground mines. A similar relation is seen in surface mines (only in seam height between 40 and 75 vs. seam height greater than 75 in.). However, RCS concentration indicated a direct relation with seam thickness both in underground mines (only in seam height ≤ 40 vs. seam height > 75 in.) and surface mines (only in seams between 40 and 75 vs. seam height > 75 in.).

Currently, RCS content in RCMD is measured from filter samples using infrared spectroscopy (NIOSH 7603 standard method) and FTIR (MSHA P-7 standard method)^53,54^. Because of the complicated tasks needed to prepare samples for centralized laboratory assessment, the significant lag time between the collection of samples and the results is generally observed. To address this issue, NIOSH has been researching instrument development to provide a near real-time measurement of quartz concentration at the end of the shift^55^. These investigations include adjusting a field FTIR instrument on a standard filter using a gravimetric sampler. Using this nondestructive method can provide immediate RCS concentration, and filters could also be analyzed in the laboratory for further dust characterization. NIOSH is developing the CPDM to directly measure RCS content by adjusting either Fourier transform infrared spectroscopy (FTIR)^56^ or X-ray fluorescence (XRF)^57^; both have been used as post-shift methods of analysis. However, there are challenges in using these techniques in real-time measurement, including an unappropriated TEOM-based filter for FTIR analysis. The CPDM filter media interferences for crystalline silica analysis while using XRF.

It is evident that more research on the physical and chemical properties of respirable dust is needed to understand the root causes of CWP incidences and its recent resurgence. Characteristics parameters such as particle size distribution (particularly in the smaller fractions enriched with minerals), mineral composition, trace element presence, particle shape, and angularity are critical parameters that need to be examined closely. To date, there has been no comprehensive study to investigate the relationship between respirable coal mine dust characterization and respiratory deposition. Many studies demonstrate that by increasing the tidal volume (whether by increment activity or through the ventilation system), a more considerable amount of dust particles will be deposited in the human respiratory system. Furthermore, the size fraction of particle deposition may differ by the breathing scenario, pause fraction, and breathing frequency for adults and young workers^29,58–60^. It is crucial to identify different constituents of RCMD to have a better understanding of the health effects. Not all RCMD particles inhaled into the miner’s respiratory tract will be deposited in the lung^61^. A portion of the inhaled RCMD will be exhaled out of the respiratory tract during exhalation. Therefore, the total amount of RCMD inhaled may not represent the RCMD actual exposure dose. This phenomenon is especially significant for submicron particles^60,62^.

## Conclusion

The rise in the prevalence of CWP in the U.S. in the mid-1990s has renewed the urge among scientists and medical researchers to look into the main underlying causes of the problem. There has been no study to investigate the effect of all mining factors on respirable silica and coal dust concentration. This study made an effort to investigate contributing factors in RCMD and RCS dust concentrations in both U.S. underground and surface coal mines. The literature is reviewed to extract reliable data and investigate gaps in previous studies. Using GEE model on MSHA database indicates relationship of different mine-related factors with dust concentration. This provides deeper insights into investigation on the resurgence of coal worker’s lung diseases. In this study, the potential reasons for increasing dust exposure in the U.S. coal mines have been investigated using a multivariable model. The results indicate significantly higher RCMD concentration in underground mines, while RCS concentration is higher in surface mines. In addition, Interior large underground mines with seams less than 40 in. show significant positive values for RCMD concentration. In comparison, medium mines with seams between 40 and 75 in. show significant RCMD exposure in surface mines, although the coefficients are relatively small. Furthermore, Appalachia small mines with seams greater than 75 in. show significant RCS exposure in both underground and surface mines. However, there are limitations within the MSHA database including inconsistency and missing data. More limitation is the efficacy of RCMD monitoring approaches which is still questionable and there has been no comprehensive study that compares mass-concentration with number-concentration based RCMD monitoring. Also, there has been no study to investigate whether RCMD mass content represents the true dose of RCMD received by a coal miner.

## Data Availability

The datasets used and/or analyzed during the current study are available from the MSHA library (https://arlweb.msha.gov/OpenGovernmentData/OGIMSHA.asp).
